# Museum of the Philadelphia Hospital

**Published:** 1860-11

**Authors:** 


MUSEUM OF THE PHILADELPHIA HOSPITAL.—After a conference with the Medical Board, the Board of Guardians adopted a resolution establishing a museum for the preservation of pathological and other specimens, and a considerable fund has already been appropriated for obtaining fixtures and materials for the basis of a cabinet. A yearly endowment will be granted, and Dr. Agnew has been appointed curator of the museum.

